# Transient receptor potential channels in *Flaviviridae* infection: A comprehensive review

**DOI:** 10.1080/19336950.2026.2665540

**Published:** 2026-05-05

**Authors:** Fuchun Jiang, Xiaoqiang Yao, Chang-Bo Zheng

**Affiliations:** a School of Pharmaceutical Science and Yunnan Key Laboratory of Pharmacology for Natural Products, Kunming Medical University, Kunming, China; b School of Biomedical Sciences and Li Ka Shing Institute of Health Science, Faculty of Medicine, The Chinese University of Hong Kong, Hong Kong, China; c Yunnan Key Laboratory of Cross-Border Infectious Disease Control and Prevention and Novel Drug Development, China

**Keywords:** Flaviviridae, transient receptor potential channels, calcium signaling, viral infection

## Abstract

Diseases caused by *Flaviviridae* viruses exert profound global health and economic burdens due to their high incidence and associated mortality. Infection by the *Flaviviridae* family often leads to severe acute or chronic illnesses, including hemorrhagic systemic diseases caused by dengue virus (DENV) and yellow fever virus, neurological complications associated with West Nile virus and Zika virus (ZIKV) infection, as well as liver damage and hepatocellular carcinoma resulting from hepatitis C virus (HCV) infection. Accumulating evidence indicates that a hallmark of *Flaviviridae* infection is to hijack calcium (Ca^2+^) signaling of the host cells, consequently facilitating viral entry, RNA replication, viral assembly, and release. Transient receptor potential (TRP) channels, a superfamily of nonselective cation channels, are central regulators of intracellular Ca^2+^ homeostasis. Recent studies reveal that TRP channels are closely involved in *Flaviviridae* infection. This comprehensive review synthesizes current findings on the role of TRP channels (e.g. TRPC, TRPV, TRPA, TRPM, and TRPML) in the pathogenesis of *Flaviviridae* viruses such as DENV, ZIKV, HCV, and Japanese encephalitis virus. Moreover, this review summarizes virus-TRP channel interaction mechanisms and discusses the potential of targeting TRP channels for host-directed antiviral therapies. This review aims to establish a conceptual framework for understanding TRP channel biology in *Flaviviridae* infection and to guide future research on antiviral strategies. Future directions should highlight the potential utility of TRP channel research for advancing anti-*Flaviviridae* therapies and clarifying their underlying mechanisms.

## GRAPHIC ABSTRACT

**Figure:**
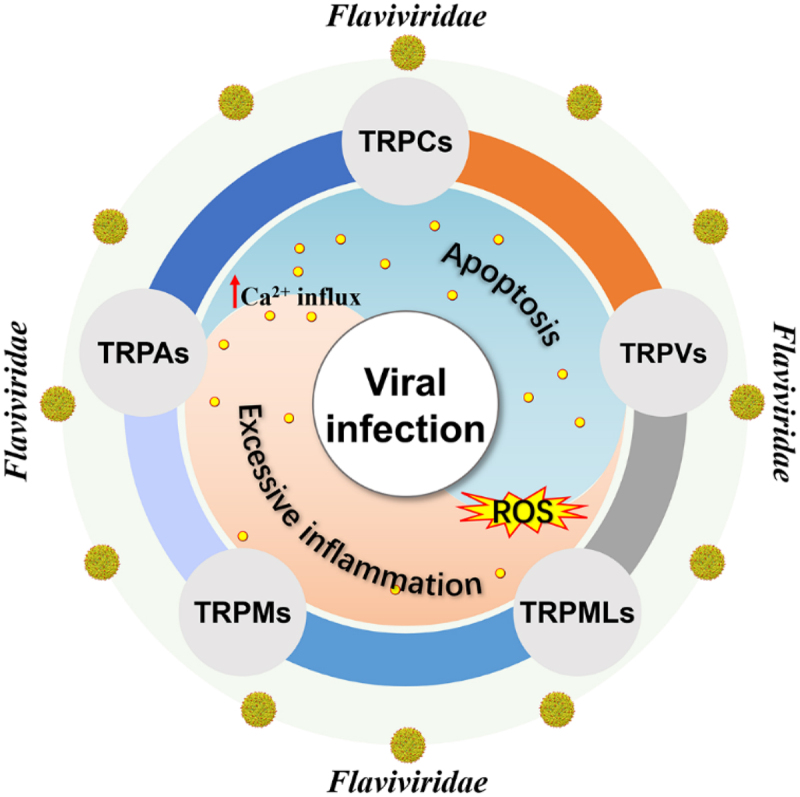

## Introduction

The *Flaviviridae* family comprises a diverse group of enveloped, single-stranded positive-sense RNA viruses that present substantial threats to global public health and animal industries [1]. The infection caused by these viruses has reached endemic levels across more than 100 countries and regions, affecting hundreds of millions of individuals and often leading to severe complications [2]. Members of the *Flavivirus* genus, such as dengue virus (DENV), Zika virus (ZIKV), yellow fever virus (YFV), and Japanese encephalitis virus (JEV), are primarily transmitted through blood-sucking arthropods (e.g. mosquitoes and ticks) [3], resulting in over 400 million human infections each year [4]. Clinical outcomes range from mild febrile illness to hemorrhagic fever (as seen with DENV and YFV) and even encephalitis associated with ZIKV and JEV infections [5]. Meanwhile, the *Hepacivirus* genus includes human hepatitis C virus (HCV), a blood-borne pathogen that chronically infects approximately 58 million people worldwide and can progress to chronic liver disease, cirrhosis, and hepatocellular carcinoma [6]. *Pestiviruses*, such as classical swine fever virus and bovine viral diarrhea virus, also cause considerable economic losses in livestock production, further highlighting the wide-ranging impact of this viral family [7]. Although effective vaccines exist for some *Flaviviruses* like JEV [8] and YFV [9], outbreaks continue to emerge due to factors such as globalization and urbanization [10], which facilitate disease vector reestablishment and human transmission. Moreover, the absence of broadly effective vaccines or FDA-approved antiviral treatments leaves nearly 3.9 billion people, largely in low-income regions, vulnerable to infection [11,12]. Therefore, a comprehensive understanding of the biological characteristics of *Flaviviruses* is urgently needed.

A defining characteristic of *Flaviviridae* infection is the strategic manipulation of host cellular signaling to support viral entry, replication, assembly, and release. Notably, these viruses often induce host cell dysfunction and disrupt intracellular calcium (Ca^2+^) homeostasis [13]. As a ubiquitous second messenger, Ca^2+^ is critical for virtually all physiological processes in the humans [14]. Over recent decades, transient receptor potential (TRP) channels, a family of polymodal cation channels with high selectivity for Ca^2+^, have attracted growing research interest due to their central role in maintaining intra- and extracellular Ca^2+^ homeostasis [15]. Previous studies have proved that dysregulation of TRP-mediated Ca^2+^ signaling, along with ensuing physiopathological alterations, acts as a key driver in the progression of viral infectious diseases. TRP channels are categorized into seven subfamilies (TRPC, TRPV, TRPM, TRPN, TRPA, TRPP, TRPML) based on structural homology, and they respond to diverse stimuli (e.g. osmotic pressure, pH, mechanical force, ligands) to maintain Ca^2+^ flux [16]. Beyond their physiological functions in sensory transduction and cellular metabolism, TRP channels are increasingly recognized as critical mediators of viral infection, functioning as “molecular gatekeepers” that pathogens co-opt or disrupt to promote their replication and spread [17]. Previous studies have confirmed that TRP subfamilies (e.g. TRPC and TRPML) promote the replication of herpes simplex virus type 1 into cells by regulating store-operated Ca^2+^ entry or endosomal Ca^2+^ release [18,19]. Similarly, TRPV4 overexpression is found to enhance the entry and replication of *Flaviviruses* such as HCV, ZIKV, and DENV via increased Ca^2+^ influx [20]. Conversely, pharmacological inhibition of TRP channels, including TRPML [21], TRPC4 [22], and TRPV1 [23], has been shown to reduce *Flavivirus* infection and dissemination. Despite these insights, a systematic analysis of the role and mechanisms of TRP channels across *Flaviviridae* infection remains lacking.

This review addresses these gaps by synthesizing existing evidence on TRP channels in *Flaviviridae* infection. We summarize the structural and functional diversity of TRP channels and discuss their contributions to the life cycles of major *Flaviviridae* pathogens. Moreover, we further explore mechanistic aspects, such as interactions between viral infection and TRP channels, and the modulation of Ca^2^ -dependent signaling cascades. Furthermore, we evaluate the therapeutic potential of targeting TRP channels for antiviral strategies. By synthesizing these findings, this review provides a cohesive framework for understanding how TRP channels affect *Flaviviridae* infection and offers directions for developing novel antiviral strategies.

## Overview of Flaviviridae structure and infection

**Figure 1.**
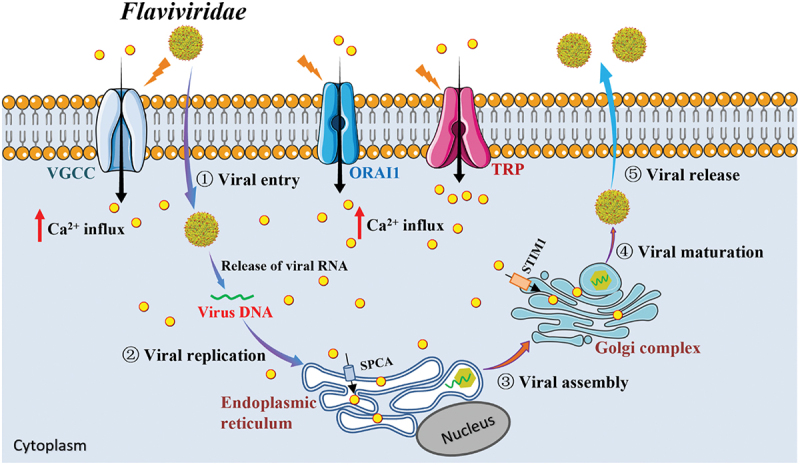
Ca^2+^ signaling involved in the life cycle of *Flaviviridae* viruses.

The *Flaviviridae* family, a group of enveloped single-stranded positive-sense RNA genome that encodes three structural proteins (including capsid (C), premembrane/membrane, and envelope (E)) and seven nonstructural proteins (e.g. NS1, NS2A, NS2B, NS3, NS4A, NS4B, and NS5) [24], encompasses various pathogens that pose significant threats to human and animal health. This family consists of four genera: *Flavivirus*, *Hepacivirus*, *Pegivirus*, and *Pestivirus* [25]. Viruses in the *Flavivirus* genus are primarily transmitted by blood-feeding arthropods (such as mosquitoes, ticks, and sandflies) and can infect humans, causing a range of clinical symptoms, including fever, headache, back pain, conjunctival congestion, rash, and severe hemorrhagic fever [26,27]. The *Flavivirus* genus contains over 70 members [28], including well-known human pathogens like ZIKV, DENV, JEV, WNV, and YFV. Another important genus within *Flaviviridae* is *Hepacivirus*, which includes the blood-borne human HCV that contributes to the occurrence and progression of liver fibrosis, cirrhosis, and hepatocellular carcinoma [29,30]. Moreover, the *Pestivirus* genus are zoonotic pathogens that impact on human health [31], which includes bovine viral diarrhea virus (BVDV), classical swine fever virus (CSFV), and border disease virus (BDV). Numerous studies have confirmed that *Flaviviridae* viruses have been detected in infected animals and humans and cause major public health concerns worldwide [32]. Functionally, viruses are adept at disrupting the Ca^2+^ homeostasis of host cells, which helps the viruses to achieve successful infections. Meanwhile, Ca^2+^ homeostasis disruption is a hallmark of viral infection [33]. As a ubiquitous second messenger, Ca^2+^ signaling participates in the regulation of a wide variety of physiological and biochemical processes, including the invasion, replication, proliferation, and release of *Flaviviridae* viruses [34,35]. Other studies have found that *Flaviviridae* infection facilitates Ca^2+^ signaling activation [36,37] and that Ca^2+^ signaling can serve as a potential therapeutic target for viral infection [38]. Herein, the functional role of Ca^2+^ signaling in *Flaviviridae* infection is summarized in Figure 1.

*Flaviviridae* viruses drive the intracellular calcium overload and endosome membrane to release the nucleocapsid (RNA genetic material). *Flaviviridae* virus replication and virion assembly occur at the endoplasmic reticulum. Immature virion is then transported to the Golgi apparatus and the maturation happens in the trans-Golgi network. Viral particle is finally released outside the cell through exocytosis pathway. VGCC: voltage-gated Ca^2+^ channels; ORAI1: orai calcium release-activated calcium modulator 1; TRP: transient receptor potential; SPCA: secretory pathway Ca^2+^-ATPase; STIM1: stromal interaction molecule.

## TRP channels

**Figure 2.**
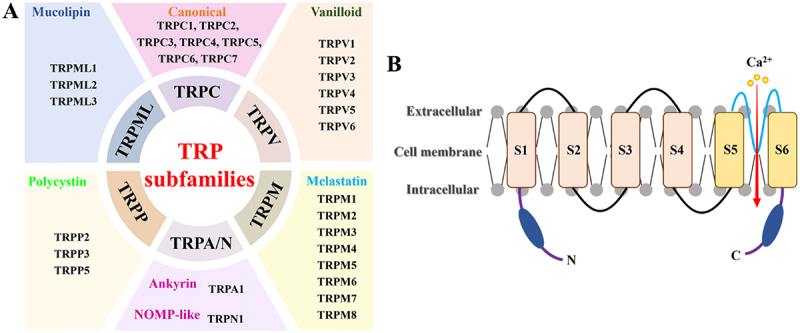
Schematic diagram of the TRP channel family and structure.

TRP channels were first discovered in *Drosophila* in 1969. As key regulators of intracellular Ca^2+^ homeostasis, TRP channels play an important role in regulating host homeostasis and various viral infections [17]. Based on the homology of amino acid sequences, mammalian TRP channels were divided into seven main subfamilies (Figure 2A): TRPC (canonical), TRPV (vanilloid), TRPM (melastatin), TRPN (non-mechanoreceptor potential C), TRPA (ankyrin), TRPP (polycystin), and TRPML (mucolipin) [39]. Structurally, TRP channels have a conserved primary structure that consists of six transmembrane domains (S1-S6), two cytoplasmic domains (NH2 and COOH termini), and a pore-forming loop located between S5 and S6 (Figure 2B).

A: members of the TRP channel family; B: TRP channel structure.

Currently, TRP channels are widely distributed in human organs, including the trigeminal nerve, lung, liver, heart, kidney, intestinal tract, and spinal nerve. Previous studies have demonstrated that the activity of TRP channels can be regulated by multiple physical and chemical factors, such as osmotic pressure, cyclic nucleotides, mechanical force, and biochemical interactions involving ligands or cellular proteins [16,40]. Numerous studies have confirmed that TRP channels have been regarded as promising new therapeutic targets for various diseases, including cardiovascular diseases [41,42], cancer [43], pain [44], liver disease [45], inflammatory bowel disease [46], lung disease [47], rheumatoid arthritis [48], *etc*. In recent years, dysregulation of TRP channel-mediated Ca^2+^ homeostasis has been identified as a critical factor for the occurrence and prognosis of viral disease [17].

## Role of TRP channels in Flaviviridae infection

**Figure 3.**
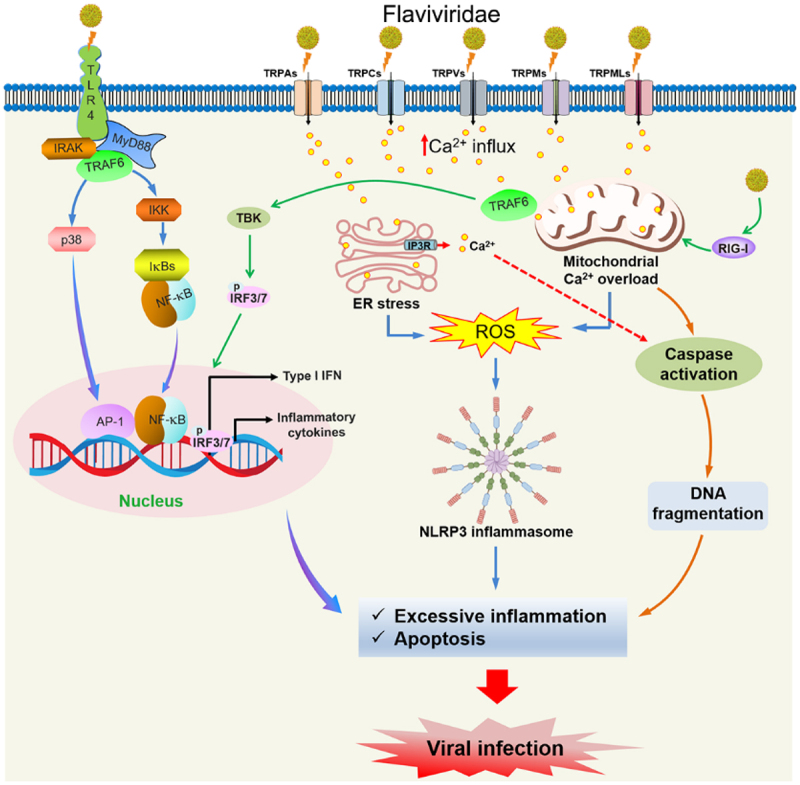
Regulatory role of TRP channel-mediated Ca^2+^ signaling in *Flaviviridae* infection.

TRP channels in host cells can be used by viruses to promote their life cycle, including entry, replication, assembly, and export [49]. Understanding the interaction between the *Flaviviridae* family and TRP channels may provide novel therapeutic targets and antiviral drugs for diseases caused by *Flaviviridae* infection. Currently, five different subfamilies of TRP channels, including TRPC, TRPV, TRPA, TRPM, and TRPML, have been reported to interact with *Flaviviridae* infection. Herein, the functional role and mechanism of TRP channels in *Flaviviridae* infection are summarized in Table 1 and Figure 3.

TRP channels are involved in the host immune and inflammatory response. During this process, TRP channels activation and Ca^2+^ influx are involved. *Flaviviridae* virus infection caused TLR4 receptor activation and promoted MyD88 and NF-κB inflammatory pathway. Moreover, TRPAs, TRPCs, TRPVs, TRPMs, and TRPMLs are involved in the activation of the NLRP3 inflammasome, as well as the caspase activation. TLR: toll-like receptor; IRAK: IL-1 receptor-associated kinase; NF-κB: nuclear factor kappa-B; AP-1: activator protein-1; IRF: interferon regulatory factor; TBK: TANK-binding kinase; TRAF6: TNF receptor-associated factor 6; RIG-I: retinoic acid-inducible gene I; ERS: endoplasmic reticulum stress.

**Table 1.** The expression and mechanisms of TRP channels in *Flaviviridae* infection.

TRP channel	Experimental model	Expression	Effect and mechanisms	Specific inhibitor/agonist	Ref.
TRPC4	ZIKV infection Cell: BHK cells Animal: A129 mice	Upregulation	Promoting the survival rate of ZIKV-infected cells and viral protein production	Inhibitor: HC-070 and KN-93	[22]
TRPV1	ZIKV infection	Upregulation	Promoting inflammation	/	[23]
TRPV4	DENV, HCV, and ZIKV infection Cell: Huh7 cells	Upregulation	Promoting viral replication	/	[20]
TRPV4	ZIKV infection Cell: Huh7 cells	Upregulation	Promoting ZIKV infectivity	Inhibitor: NSC151066	[50]
TRPA1	Yellow fever mosquito *Aedes aegypti* Cell: Oocytes Animal: Mosquito	Downregulation	Inhibiting the life cycle of YFV	Agonist: a bark extract of *Cinnamosma fragrans*	[51]
TRPM5	HCV infection	Upregulation	Promoting hepatic fibrosis progression in non-cirrhotic HCV-infected patients	/	[52]
TRPMLs	DENV2 and ZIKV infection Cell: A549 cells	Downregulation	Inhibiting viral entry and replication	Agonist: ML-SA1 and SN-2	[21,53]
TRPMLs	JEV infection Cell: A549 cells	Upregulation	Promoting JEV entry, egress, and infection	Inhibitor: berbamine	[54]
TRPML	*Flavivirus* (DENV, JEV, ZIKV) Cell: A549 cells	Upregulation	Promoting *flavivirus* entry and replication	Inhibitor: bis-benzylisoquinoline alkaloids	[55]
TRPML2	ZIKV infection Cell: A549 cells	Downregulation	Inhibiting ZIKV infection	Agonist: ML2-AS1	[56]

### TRPC

As the earliest discovered members of the TRP family, TRPC channels are nonselective cation channels with molecular weights ranging from several tens to hundreds of kilodaltons [57]. Based on amino acid similarity, mammalian TRPC channels are classified into four subgroups: TRPC1, TRPC2, TRPC3/6/7, and TRPC4/5 [58]. Viruses can hijack TRPC channels in host cells to generate a favorable environment for viral entry. For example, knockdown of TRPC1 reduces herpes simplex virus type 1 (HSV-1) infection by enhancing Ca^2+^ influx and ameliorated HSV-1-induced ocular abnormality and morbidity in mice [19]. Of note, TRPC1 has been reported to be a component of the store-operated channels [59,60], which facilitates viral infection and pathogenesis by accelerating Ca^2+^ influx [61,62]. A recent study showed that suppression of TRPC4 inhibits the proliferation and spread of ZIKV and prevented ZIKV-associated seizures and death [22]. Collectively, TRPC channels are known to be involved in ZIKV infection, but their functional role and underlying mechanism in *Flaviviridae* infection is not fully understood.

### TRPV

The TRPV channels belong to the vanilloid subtype of the TRP family, which comprises six members, namely TRPV1, TRPV2, TRPV3, TRPV4, TRPV5, and TRPV6. TRPV channels play a crucial regulatory role in the development of neuropathic pain [44,63], contributing to their function as sensors for diverse pain stimuli (e.g. heat, pressure, and pH). A recent study reported that TRPV1 ablation or antagonism ameliorates Chikungunya virus (CHIKV)-induced pain [64]. In addition, TRPV1 expression is enhanced in airway cells after respiratory virus infection [65]. Existing studies have reported that TRPV channels can regulate viral infection and represent a potential target for antiviral therapy. For example, TRPV1 deficiency reduces influenza A virus [66] replication. Deletion of TRPV2 restricts the replication and spread of Vesicular stomatitis virus [67] and HSV-2 [68] by promoting Ca^2+^ influx, as well as reduces inflammation induced by SARS-CoV-2 infection [69]. Zhang et al. [70] showed that overexpression of TRPV4 promotes HBV entry, replication, and capsid assembly. Another study showed that TRPV4 can serve as an effective biomarker for designing antiviral agents [50]. In the *Flaviviridae* family, inhibition of TRPV4 suppresses infectivity of DENV, HCV, and ZIKV by modulating Ca^2+^ influx [20]. Bhandari et al. [23] summarized that TRPV1 may serve as a novel therapeutic target for ZIKV-induced neurological complications. Mechanistically, targeting TRPV channels with agonists or antagonists can relieve various viral infection-induced inflammation. Vanwalscappel et al. [71] showed that toll-like receptor agonist R848 treatment restricts ZIKV replication in macrophages by enhancing antiviral protein viperin levels. Taken together, TRPV channels play an important role in the entry and replication of *Flaviviridae*.

### TRPA

TRPA1, the only member of the TRPA channels in mammals, is known for its activation by various chemical irritants and viral infection [72]. Numerous studies have confirmed that TRPA1 expression is increased in host cells after viral infection, such as human rhinovirus [73] and respiratory viruses [65]. Meanwhile, reactive oxygen species (ROS) and inflammation, which often increase during viral infection, can activate TRPA1 [74,75]. Another study reported that mosquito saliva contains metabolites (e.g. sphingomyelins) that promotes *Flavivirus* transmission and enhances TRPA1 expression by suppressing host endoplasmic reticulum-associated degradation [76,77]. Meanwhile, inhibition of TRPA1 by *Aedes aegypti* salivary gland extract contributes to ameliorating acute itching [78]. Previous studies have proved that *Flaviviridae* infection increases mitochondrial ROS and inflammation [79,80], which can activate TRPA1, leading to Ca^2+^ overload and an enhanced viral genome translation. Notably, knockdown of TRPA1 reduces inflammation during Chikungunya virus infection in macrophages [81]. Bhandari et al. [23] proved that upregulation of TRPA1 promotes neurological complications caused by ZIKV infection. Collectively, TRPA1 may involve in *Flaviviridae* pathogenesis, but its specific mechanism still needs to be further explored.

### TRPM

The TRPM channels belong to the melastatin group of TRP family, which comprises of eight members, including TRPM1-8. Out of these, TRPM2 and TRPM7 are often linked to responses to oxidative stress and inflammation [82], both of which are hallmarks of *Flaviviridae* infection. A recent study confirmed that TRPM2 deficiency attenuates H9N2 virus-induced lung injury by reducing oxidative stress, mitochondrial dysfunction, and inflammation [83]. Conversely, overexpression of TRMP2 enhances hepatitis B virus replication through autophagy induction [84]. In HIV infection, TRPM4 overexpression is found to exacerbate neuroinflammation and neuronal death [85]. A polymorphism in TRPM5 (rs886277) has been linked to HCV-related liver fibrosis and cirrhosis [52]. Additionally, TRMP8 has been identified as a potential target for controlling virus entry and replication [73]. Despite these findings, no direct evidence currently links TRPM channels to *Flaviviridae* entry or replication, representing a significant knowledge gap.

### TRPML

The TRPML channels belong to the mucolipin subgroup of the TRP family, which consist of three members, namely TRPML1, TRPML2, and TRPML3. Among them, TRPML2 and TRPML3 have been confirmed to regulate diverse viral infections [86]. For instance, ectopically expressed TRPML2 promotes influenza A virus, DENV, YFV, and ZIKV entry and replication [87]. Similarly, TRPML3 overexpression facilitates influenza A virus infection [87]. Conversely, TRPML2 activator (ML2-SA1) treatment impairs ZIKV replication and hepatitis E virus infection [56]. Moreover, overexpression of TRPML2 activates innate immune responses, as evidenced by promoting macrophage and neutrophil migration and CCL2 secretion [88,89]. In summary, these results indicate that TRPML channels may be a promising target for combating *Flaviviridae* infection.

## Therapeutic potential of TRP channel agonists and antagonists in Flaviviridae infection

Given the critical role of TRP channels in mediating *Flaviviridae* virus-induced abnormal intracellular Ca^2+^ influx and viral life-cycle, targeting TRP channels with agonists or antagonists has emerged as a promising antiviral strategy. For example, the Ca^2+^ channel blocker lacidipine exhibits an antiviral effect against ZIKV replication by disturbing the subcellular distribution of free cholesterol and neutral lipids in both Vero cells and neural progenitor cells [90]. Similarly, bepridil, also a Ca^2+^ channel blocker, has broad-spectrum anti-filovirus activity [91]. Moreover, TRP channel antagonists have been shown to have high efficacy in inhibiting viral replication by restoring host Ca^2+^ homeostasis or blocking virus-dependent TRP activation. For instance, the nonselective TRPC antagonist SKF96365 suppresses DENV replication in human hepatic cells by inactivation of the store-operated Ca^2+^ entry pathway [92]. Treatment with the selective TRPV4 inhibitor GSK2193874 not only reduces viral infectivity but also blocks Ca^2+^influx [68]. Doñate-Macian et al. [50] reported that the TRPV4 antagonist (compound NSC151066) reduces ZIKV replication with an IC_50_ of 145 nM, which exhibits a potency similar to that of HC067047. Another study showed that a TRPV4 inhibitor (HC067047) suppresses the infectivity of DENV, HCV, and ZIKV in Huh7 cells by enhancing Ca^2+^ influx [20]. Inversely, treatment with a specific TRPML agonist (ML-SA1) inhibits DENV and ZIKV infection by enhancing lysosome acidification and protease activity, leading to viral degradation [21]. Similarly, a TRPA1 activator cinnamodial is toxic and repellent to the yellow fever mosquito *Aedes aegypti* [51]. Recently, natural compounds such as bis-benzylisoquinoline alkaloids have been shown to inhibit flavivirus entry and replication by blocking TRPML channels, thereby disrupting endolysosomal trafficking and autophagy [55]. Huang et al. [54] further reported that berbamine inhibits JEV infection through suppression of TRPML-mediated endolysosomal trafficking of the low-density lipoprotein receptor. However, there are currently no TRP-targeting drugs approved for the clinical treatment of viral infection, especially those caused by *Flaviviridae*.

Translating TRP channel antagonists or agonists into clinical use remains particularly challenging. One major concern is the widespread tissue distribution of TRP channels, which increases the risk of off-target effects. For instance, TRPV1 agonists might cause pain or hyperthermia. Moreover, since different viruses depend on specific TRP channels (e.g. HCV utilizes TRPC, whereas CSFV involves TRPML2), developing subgroup-selective modulators is essential, rather than broadly inhibiting TRP channels. Future efforts should prioritize improving the selectivity of TRP channel modulators and assessing their synergistic potential with established antiviral agents, such as direct-acting antivirals against *Flaviviridae*, to achieve better treatment outcomes.

In summary, preclinical evidence strongly suggests that both TRP channel agonists and antagonists represent promising candidates for antiviral therapy against *Flaviviridae* infection. By targeting host cell Ca^2+^ signaling instead of viral proteins, these compounds may also decrease the likelihood of viral resistance development, positioning them as a valuable complement to existing antiviral strategies.

## Conclusions and future perspectives

TRP channels, especially TRPC, TRPV, TRPA, and TRPM subtypes, offer distinct advantages in modulating immune responses and mitigating virus-induced pathological damage. Meanwhile, TRPML channels act as a double-edged sword, both facilitating viral replication and exhibiting antiviral effects. Based on comprehensive research, TRPC, TRPV, TRPA, TRPM, and TRPML channels are all considered promising therapeutic targets for preventing or treating *Flaviviridae* infection.

Although the role of TRP channels in viral infection, including those caused by *Flaviviridae*, has been confirmed by relevant studies, several key aspects require in-depth exploration. Firstly, mechanistic understanding, including the molecular mechanism of TRP channel activation remains unclear. The regulatory effects of different localizations of TRP channels on the entry, replication, and release of *Flaviviridae* viruses also need further clarification. Secondly, clinical translation issues need to be further investigated. The widespread expression of TRP channels and their multiple biological functions pose challenges for drug development, and target-related side effects often limit clinical applications. Furthermore, most studies on TRP channels in *Flaviviridae* infection rely on *in vitro* models or rodent models. Therefore, future research should focus on three directions: (1) employing structural biology and proteomics to map virus-TRP channels interaction interfaces; (2) developing humanized models (such as organoids) to validate TRP-targeted therapies; and (3) exploring combination treatment strategies (e.g. TRP channel modulators with direct-acting antivirals) to enhance efficacy and reduce resistance. Addressing these research gaps will not only deepen our understanding of *Flaviviridae* pathogenesis but also accelerate the development of TRP channel-based therapy strategies to combat these globally impactful pathogens.

In summary, this review synthesizes the current understanding of TRP channels in *Flaviviridae* infection, highlighting their multifaceted roles as key mediators of viral hijacking of host Ca^2 +^ homeostasis. Preclinical studies have confirmed that treatment of TRP channel agonists and antagonists can restore host Ca^2+^ balance and inhibit viral entry and replication, underscoring their potential as antiviral agents.

## Data Availability

Data sharing is not applicable to this article as no data were created or analyzed in this study.

## References

[1] Sadanandane C, Gokhale MD, Elango A, et al. Prevalence and spatial distribution of ixodid tick populations in the forest fringes of Western Ghats reported with human cases of Kyasanur forest disease and monkey deaths in South India. Exp Appl Acarol. 2018;75(1):135–12. doi: 10.1007/s10493-018-0223-5 29594846

[2] Centofanti SM, Eyre NS. Live-cell imaging of Flaviviridae family virus infections: progress and challenges. Viruses. 2025;17(6):847. doi: 10.3390/v17060847 40573438 PMC12197577

[3] Mackenzie JS, Gubler DJ, Petersen LR. Emerging flaviviruses: the spread and resurgence of Japanese encephalitis, West Nile and dengue viruses. Nat Med. 2004;10(12 Suppl):S98–109. doi: 10.1038/nm1144 15577938

[4] Bhatt S, Gething PW, Brady OJ, et al. The global distribution and burden of dengue. Nature. 2013;496(7446):504–507. doi: 10.1038/nature12060 23563266 PMC3651993

[5] Tripathi A, Chauhan S, Khasa R. A comprehensive review of the development and therapeutic use of antivirals in flavivirus infection. Viruses. 2025;17(1):74. doi: 10.3390/v17010074 39861863 PMC11769230

[6] Fleurence RL, Alter HJ, Collins FS, et al. Global elimination of hepatitis C virus. Annu Rev Med. 2025;76(1):29–41. doi: 10.1146/annurev-med-050223-111239 39485830

[7] Guidoum KA, Benallou B, Pailler L, et al. Ruminant pestiviruses in North Africa. Prev Vet Med. 2020;184:105156. doi: 10.1016/j.prevetmed.2020.105156 33007610

[8] Gong J, Duan X, Ge Z. Molecular mechanisms of Japanese encephalitis virus infection and advances in vaccine research. Microb Pathog. 2025;201:107397. doi: 10.1016/j.micpath.2025.107397 39983879

[9] Tosta SFO, Passos MS, Kato R, et al. Multi-epitope based vaccine against yellow fever virus applying immunoinformatics approaches. J Biomol Struct Dyn. 2021;39(1):219–235. doi: 10.1080/07391102.2019.1707120 31854239

[10] Baker RE, Mahmud AS, Miller IF, et al. Infectious disease in an era of global change. Nat Rev Microbiol. 2022;20(4):193–205. doi: 10.1038/s41579-021-00639-z 34646006 PMC8513385

[11] Kraemer MUG, Rc R, Brady OJ, et al. Past and future spread of the arbovirus vectors Aedes aegypti and Aedes albopictus. Nat Microbiol. 2019;4(5):854–863. doi: 10.1038/s41564-019-0376-y 30833735 PMC6522366

[12] Wu K, Fan S, Zou L, et al. Molecular events occurring in lipophagy and its regulation in Flaviviridae infection. Front Microbiol. 2021;12:651952. doi: 10.3389/fmicb.2021.651952 34093468 PMC8175637

[13] Hyser JM, Estes MK. Pathophysiological consequences of calcium-conducting viroporins. Annu Rev Virol. 2015;2(1):473–496. doi: 10.1146/annurev-virology-100114-054846 26958925 PMC6538290

[14] Bean B. Calcium channels. Gating for the physiologist. Nature. 1990;348(6298):192–193. doi: 10.1038/348192a0 1978254

[15] Wu LJ, Sweet TB, Clapham DE. International Union of Basic and Clinical Pharmacology. LXXVI. Current progress in the mammalian TRP ion channel family. Pharmacol Rev. 2010;62(3):381–404. doi: 10.1124/pr.110.002725 20716668 PMC2964900

[16] Clapham DE. Trp channels as cellular sensors. Nature. 2003;426(6966):517–524. doi: 10.1038/nature02196 14654832

[17] Qi WH, Tang N, Zhao ZJ, et al. Transient receptor potential channels in viral infectious diseases: biological characteristics and regulatory mechanisms. J Adv Res. 2024;S2090-1232(24):00541–1. doi: 10.1016/j.jare.2024.11.022 PMC12793757 39551130

[18] Gunaratne GS, Marchant JS. The ins and outs of virus trafficking through acidic Ca(2+) stores. Cell Calcium. 2022;102:102528. doi: 10.1016/j.ceca.2022.102528 35033909 PMC8860173

[19] He D, Mao A, Li Y, et al. Trpc1 participates in the HSV-1 infection process by facilitating viral entry. Sci Adv. 2020;6(12):eaaz3367. doi: 10.1126/sciadv.aaz3367 32206724 PMC7080438

[20] Doñate-Macián P, Jungfleisch J, Pérez-Vilaró G, et al. The TRPV4 channel links calcium influx to DDX3X activity and viral infectivity. Nat Commun. 2018;9(1):2307. doi: 10.1038/s41467-018-04776-7 29899501 PMC5998047

[21] Xia Z, Wang L, Li S, et al. Ml-sa1, a selective TRPML agonist, inhibits DENV2 and ZIKV by promoting lysosomal acidification and protease activity. Antiviral Res. 2020;182:104922. doi: 10.1016/j.antiviral.2020.104922 32858116

[22] Chen X, Yan Y, Liu Z, et al. In vitro and in vivo inhibition of the host TRPC4 channel attenuates Zika virus infection. EMBO Mol Med. 2024;16(8):1817–1839. doi: 10.1038/s44321-024-00103-4 39009885 PMC11319825

[23] Bhandari R, Gupta R, Vashishth A, et al. Transient receptor potential vanilloid 1 (TRPV1) as a plausible novel therapeutic target for treating neurological complications in ZikaVirus. Med Hypotheses. 2021;156:110685. doi: 10.1016/j.mehy.2021.110685 34592564

[24] Van Leur Sw, Heunis T, Munnur D, et al. Pathogenesis and virulence of flavivirus infections. Virulence. 2021;12(1):2814–2838. doi: 10.1080/21505594.2021.1996059 34696709 PMC8632085

[25] Barrows NJ, Campos RK, Liao KC, et al. Biochemistry and molecular biology of flaviviruses. Chem Rev. 2018;118(8):4448–4482. doi: 10.1021/acs.chemrev.7b00719 29652486 PMC5937540

[26] Carbaugh DL, Lazear HM. Flavivirus envelope protein glycosylation: impacts on viral infection and pathogenesis. J Virol. 2020;94(11):e00104–20. doi: 10.1128/jvi.00104-20 32161171 PMC7269438

[27] Van Herreweghe M, De Bruyne T, Hermans N, et al. Clinical relevance of oxidative stress biomarkers in human flavivirus infections as predictors of disease progression and severity. Rev Med Virol. 2024;34(6):e70007. doi: 10.1002/rmv.70007 39532693

[28] Jablunovsky A, Jose J. The dynamic landscape of capsid proteins and viral RNA interactions in Flavivirus genome packaging and virus assembly. Pathogens. 2024;13(2):120. doi: 10.3390/pathogens13020120 38392858 PMC10893219

[29] Spearman CW, Dusheiko GM, Hellard M, et al. Hepatitis C. Lancet. 2019;394(10207):1451–1466. doi: 10.1016/s0140-6736(19)32320-7 31631857

[30] Martinez MA, Franco S. Therapy implications of hepatitis C virus genetic diversity. Viruses. 2020;13(1):41. doi: 10.3390/v13010041 33383891 PMC7824680

[31] Marano G, Franchini M, Farina B, et al. The human pegivirus: a new name for an “ancient” virus. Can transfusion medicine come up with something new? Acta Virol. 2017;61(4):401–412. doi: 10.4149/av_2017_402 29186957

[32] Ramezannia Z, Shamekh A, Bannazadeh Baghi H. Crispr-Cas system to discover host-virus interactions in Flaviviridae. Virol J. 2023;20(1):247. doi: 10.1186/s12985-023-02216-7 37891676 PMC10605781

[33] Kumar PS, Radhakrishnan A, Mukherjee T, et al. Understanding the role of Ca(2+) via transient receptor potential (TRP) channel in viral infection: implications in developing future antiviral strategies. Virus Res. 2023;323:198992. doi: 10.1016/j.virusres.2022.198992 36309316 PMC10194134

[34] Chang CY, Wu CC, Tzeng CY, et al. Nmda receptor blockade attenuates Japanese encephalitis virus infection-induced microglia activation. J Neuroinflammation. 2024;21(1):291. doi: 10.1186/s12974-024-03288-0 39511597 PMC11545997

[35] Negash AA, Olson RM, Griffin S, et al. Modulation of calcium signaling pathway by hepatitis C virus core protein stimulates NLRP3 inflammasome activation. PLoS Pathog. 2019;15(2):e1007593. doi: 10.1371/journal.ppat.1007593 30811485 PMC6392285

[36] Scherbik SV, Brinton MA. Virus-induced Ca^2+^ influx extends survival of West Nile virus-infected cells. J Virol. 2010;84(17):8721–8731. doi: 10.1128/jvi.00144-10 20538858 PMC2918993

[37] Devi P, Punga T, Bergqvist A. Activation of the Ca(2+)/NFAT pathway by assembly of hepatitis C virus core protein into nucleocapsid-like particles. Viruses. 2022;14(4):761. doi: 10.3390/v14040761 35458491 PMC9031069

[38] Qu Y, Sun Y, Yang Z, et al. Calcium ions signaling: targets for attack and utilization by viruses. Front Microbiol. 2022;13:889374. doi: 10.3389/fmicb.2022.889374 35859744 PMC9289559

[39] Li H. Trp channel classification. Adv Exp Med Biol. 2017;976:1–8. doi: 10.1007/978-94-024-1088-4_1 28508308

[40] Minke B, Cook B. Trp channel proteins and signal transduction. Physiol Rev. 2002;82(2):429–472. doi: 10.1152/physrev.00001.2002 11917094

[41] Ding Q, Liu X, Qi Y, et al. Trpa1 promotes the maturation of embryonic stem cell-derived cardiomyocytes by regulating mitochondrial biogenesis and dynamics. STEM Cell Res Ther. 2023;14(1):158. doi: 10.1186/s13287-023-03388-3 37287081 PMC10249273

[42] Zhao Q, Li J, Ko WH, et al. Trpm2 promotes autophagic degradation in vascular smooth muscle cells. Sci Rep. 2020;10(1):20719. doi: 10.1038/s41598-020-77620-y 33244095 PMC7693237

[43] Zhong T, Zhang W, Guo H, et al. The regulatory and modulatory roles of TRP family channels in malignant tumors and relevant therapeutic strategies. Acta Pharm Sin B. 2022;12(4):1761–1780. doi: 10.1016/j.apsb.2021.11.001 35847486 PMC9279634

[44] Koivisto AP, Voets T, Iadarola MJ, et al. Targeting TRP channels for pain relief: a review of current evidence from bench to bedside. Curr Opin Pharmacol. 2024;75:102447. doi: 10.1016/j.coph.2024.102447 38471384

[45] Wang W, Liu P, Zhang Y, et al. Expression and functions of transient receptor potential channels in liver diseases. Acta Pharm Sin B. 2023;13(2):445–459. doi: 10.1016/j.apsb.2022.09.005 36873177 PMC9978971

[46] Du Y, Chen J, Shen L, et al. Trp channels in inflammatory bowel disease: potential therapeutic targets. Biochem Pharmacol. 2022;203:115195. doi: 10.1016/j.bcp.2022.115195 35917870

[47] Müller I, Alt P, Rajan S, et al. Transient receptor potential (TRP) channels in airway toxicity and disease: an update. Cells. 2022;11(18):2907. doi: 10.3390/cells11182907 36139480 PMC9497104

[48] Niu M, Zhao F, Chen R, et al. The transient receptor potential channels in rheumatoid arthritis: need to pay more attention. Front Immunol. 2023;14:1127277. doi: 10.3389/fimmu.2023.1127277 36926330 PMC10013686

[49] Jones PE, Pérez-Segura C, Bryer AJ, et al. Molecular dynamics of the viral life cycle: progress and prospects. Curr Opin Virol. 2021;50:128–138. doi: 10.1016/j.coviro.2021.08.003 34464843 PMC8651149

[50] Doñate-Macian P, Duarte Y, Rubio-Moscardo F, et al. Structural determinants of TRPV4 inhibition and identification of new antagonists with antiviral activity. Br J Pharmacol. 2022;179(14):3576–3591. doi: 10.1111/bph.15267 32959389 PMC9291951

[51] Inocente EA, Shaya M, Acosta N, et al. A natural agonist of mosquito TRPA1 from the medicinal plant *Cinnamosma fragrans* that is toxic, antifeedant, and repellent to the yellow fever mosquito Aedes aegypti. PLOS Negl Trop Dis. 2018;12(2):e0006265. doi: 10.1371/journal.pntd.0006265 29425195 PMC5823474

[52] Resino S, Fernández-Rodríguez A, Pineda-Tenor D, et al. Trpm5 rs886277 polymorphism predicts hepatic fibrosis progression in non-cirrhotic HCV-infected patients. J Clin Med. 2021;10(3):483. doi: 10.3390/jcm10030483 33525598 PMC7865714

[53] Xia Z, Ren Y, Li S, et al. Ml-sa1 and sn-2 inhibit endocytosed viruses through regulating TRPML channel expression and activity. Antiviral Res. 2021;195:105193. doi: 10.1016/j.antiviral.2021.105193 34687820

[54] Huang L, Li H, Ye Z, et al. Berbamine inhibits Japanese encephalitis virus (JEV) infection by compromising TPRMLs-mediated endolysosomal trafficking of low-density lipoprotein receptor (LDLR). Emerg Microbes Infect. 2021;10(1):1257–1271. doi: 10.1080/22221751.2021.1941276 34102949 PMC8238074

[55] Huang L, Liu L, Zhu J, et al. Bis-benzylisoquinoline alkaloids inhibit flavivirus entry and replication by compromising endolysosomal trafficking and autophagy. Virol Sin. 2024;39(6):892–908. doi: 10.1016/j.virs.2024.09.001 39251138 PMC11738800

[56] Schwickert KK, Glitscher M, Bender D, et al. Zika virus replication is impaired by a selective agonist of the TRPML2 ion channel. Antiviral Res. 2024;228:105940. doi: 10.1016/j.antiviral.2024.105940 38901736

[57] Latorre R, Zaelzer C, Brauchi S. Structure-functional intimacies of transient receptor potential channels. Q Rev Biophys. 2009;42(3):201–246. doi: 10.1017/s0033583509990072 20025796

[58] Wang H, Cheng X, Tian J, et al. Trpc channels: structure, function, regulation and recent advances in small molecular probes. Pharmacol Ther. 2020;209:107497. doi: 10.1016/j.pharmthera.2020.107497 32004513 PMC7183440

[59] Salido GM, Sage SO, Rosado JA. Trpc channels and store-operated Ca(2+) entry. Biochim Biophys Acta. 2009;1793(2):223–230. doi: 10.1016/j.bbamcr.2008.11.001 19061922

[60] Ambudkar IS, de Souza Lb, Ong HL, et al. Trpc1, Orai1, and Stim1 in SOCE: friends in tight spaces. Cell Calcium. 2017;63:33–39. doi: 10.1016/j.ceca.2016.12.009 28089266 PMC5466534

[61] Casciano JC, Duchemin NJ, Lamontagne RJ, et al. Hepatitis B virus modulates store-operated calcium entry to enhance viral replication in primary hepatocytes. PLOS ONE. 2017;12(2):e0168328. doi: 10.1371/journal.pone.0168328 28151934 PMC5289456

[62] Dellis O, Arbabian A, Papp B, et al. Epstein-Barr virus latent membrane protein 1 increases calcium influx through store-operated channels in B lymphoid cells. J Biol Chem. 2011;286(21):18583–18592. doi: 10.1074/jbc.M111.222257 21454636 PMC3099674

[63] Satheesh NJ, Uehara Y, Fedotova J, et al. Trpv currents and their role in the nociception and neuroplasticity. Neuropeptides. 2016;57:1–8. doi: 10.1016/j.npep.2016.01.003 26825374

[64] Da Silva Lcm, dos Santos Maia AC, de Sousa Ncf, et al. Chikungunya particle and RNA induce mechanical and heat hypersensitivities in a TRPV1-dependent manner. Biomolecules. 2025;15(2):171. doi: 10.3390/biom15020171 40001474 PMC11853433

[65] Omar S, Clarke R, Abdullah H, et al. Respiratory virus infection up-regulates TRPV1, TRPA1 and ASICS3 receptors on airway cells. PLOS ONE. 2017;12(2):e0171681. doi: 10.1371/journal.pone.0171681 28187208 PMC5302416

[66] Murakami D, Kono M, Sakatani H, et al. Inhibition of transient receptor potential vanilloid 1 reduces shedding and transmission during *Streptococcus pneumoniae* co-infection with influenza. Infect Immun. 2024;92(10):e0014624. doi: 10.1128/iai.00146-24 39109830 PMC11475660

[67] Guo YY, Gao Y, Hu YR, et al. The transient receptor potential vanilloid 2 (TRPV2) channel facilitates virus infection through the Ca(2+) -LRMDA axis in myeloid cells. Adv Sci (Weinh). 2022;9(34):e2202857. doi: 10.1002/advs.202202857 36261399 PMC9731701

[68] Jiang P, Li SS, Xu XF, et al. Trpv4 channel is involved in HSV-2 infection in human vaginal epithelial cells through triggering Ca(2+) oscillation. Acta Pharmacol Sin. 2023;44(4):811–821. doi: 10.1038/s41401-022-00975-7 36151392 PMC10042832

[69] Xu J, Yang Y, Hou Z, et al. Trpv2-spike protein interaction mediates the entry of SARS-CoV-2 into macrophages in febrile conditions. Theranostics. 2021;11(15):7379–7390. doi: 10.7150/thno.58781 34158856 PMC8210595

[70] Zhang Y, Yuan X, Wang J, et al. Trpv4 promotes HBV replication and capsid assembly via methylation modification of H3K4 and HBc ubiquitin. J Med Virol. 2024;96(4):e29510. doi: 10.1002/jmv.29510 38573018

[71] Vanwalscappel B, Tada T, Landau NR. Toll-like receptor agonist R848 blocks Zika virus replication by inducing the antiviral protein viperin. Virology. 2018;522:199–208. doi: 10.1016/j.virol.2018.07.014 30036788 PMC6130814

[72] Naert R, López-Requena A, Talavera K. Trpa1 expression and pathophysiology in immune cells. Int J Mol Sci. 2021;22(21):11460. doi: 10.3390/ijms222111460 34768891 PMC8583806

[73] Abdullah H, Heaney LG, Cosby SL, et al. Rhinovirus upregulates transient receptor potential channels in a human neuronal cell line: implications for respiratory virus-induced cough reflex sensitivity. Thorax. 2014;69(1):46–54. doi: 10.1136/thoraxjnl-2013-203894 24002057

[74] Stanford KR, Hadley SH, Barannikov I, et al. Antimycin A-induced mitochondrial dysfunction activates vagal sensory neurons via ROS-dependent activation of TRPA1 and ROS-independent activation of TRPV1. Brain Res. 2019;1715:94–105. doi: 10.1016/j.brainres.2019.03.029 30914247 PMC6500470

[75] Yang Z, Liang Y, Wu C, et al. Kemin capsule ameliorates post-infectious cough by modulating the PI3K/AKT signaling pathway and TRPA1/TRPV1 channels. J Ethnopharmacol. 2025;337(Pt 1):118837. doi: 10.1016/j.jep.2024.118837 39306207

[76] Medkour H, Pruvost L, Miot EF, et al. Sphingomyelins in mosquito saliva reconfigure skin lipidome to promote viral protein levels and enhance transmission of flaviviruses. Cell Metab. 2025;37(7):1601–1614.e11. doi: 10.1016/j.cmet.2025.05.015 40543501

[77] Derouiche S, Li T, Sakai Y, et al. Inhibition of transient receptor potential vanilloid 1 and transient receptor potential ankyrin 1 by mosquito and mouse saliva. Pain. 2022;163(2):299–307. doi: 10.1097/j.pain.0000000000002337 33990108 PMC8756345

[78] Cerqueira ARA, Rodrigues L, Coavoy-Sánchez SA, et al. Aedes aegypti salivary gland extract alleviates acute itching by blocking TRPA1 channels. Front Physiol. 2023;14:1055706. doi: 10.3389/fphys.2023.1055706 37441000 PMC10333701

[79] Valadão AL, Aguiar RS, de Arruda LB. Interplay between inflammation and cellular stress triggered by Flaviviridae viruses. Front Microbiol. 2016;7:1233. doi: 10.3389/fmicb.2016.01233 27610098 PMC4996823

[80] Losarwar S, Pancholi B, Babu R, et al. Mitochondria-dependent innate immunity: a potential therapeutic target in flavivirus infection. Int Immunopharmacol. 2025;154:114551. doi: 10.1016/j.intimp.2025.114551 40158432

[81] Sanjai Kumar P, Nayak TK, Mahish C, et al. Inhibition of transient receptor potential vanilloid 1 (TRPV1) channel regulates chikungunya virus infection in macrophages. Arch Virol. 2021;166(1):139–155. doi: 10.1007/s00705-020-04852-8 33125586

[82] Piciu F, Balas M, Badea MA, et al. Trp channels in tumoral processes mediated by oxidative stress and inflammation. Antioxid (Basel). 2023;12(7):1327. doi: 10.3390/antiox12071327 PMC10376197 37507867

[83] Li L, Xu J, Yuan J, et al. Trpm2 deficiency ameliorated H9N2 influenza virus-induced acute lung injury in mice. Microb Pathog. 2025;199:107183. doi: 10.1016/j.micpath.2024.107183 39615704

[84] Chen L, Zhu L, Lu X, et al. Trpm2 regulates autophagy to participate in hepatitis B virus replication. J Viral Hepat. 2022;29(8):627–636. doi: 10.1111/jvh.13710 35633088

[85] Li G, Makar T, Gerzanich V, et al. HIV-1 vpr-induced proinflammatory response and apoptosis are mediated through the Sur1-Trpm4 channel in astrocytes. Mbio. 2020;11(6):e02939–20. doi: 10.1128/mBio.02939-20 33293383 PMC8534293

[86] Santoni G, Morelli MB, Amantini C, et al. Involvement of the TRPML mucolipin channels in viral infections and anti-viral innate immune responses. Front Immunol. 2020;11:739. doi: 10.3389/fimmu.2020.00739 32425938 PMC7212413

[87] Rinkenberger N, Schoggins JW. Mucolipin-2 cation channel increases trafficking efficiency of endocytosed viruses. Mbio. 2018;9(1):e02314–17. doi: 10.1128/mBio.02314-17 29382735 PMC5790917

[88] Sun L, Hua Y, Vergarajauregui S, et al. Novel role of TRPML2 in the regulation of the innate immune response. J Immunol. 2015;195(10):4922–4932. doi: 10.4049/jimmunol.1500163 26432893 PMC4637233

[89] Galione A, Davis LC. Revealing the secrets of secretion. Elife. 2018;7:e43512. doi: 10.7554/eLife.43512 30499445 PMC6269119

[90] Bezemer B, van Cleef KWR, Overheul GJ, et al. The calcium channel inhibitor lacidipine inhibits Zika virus replication in neural progenitor cells. Antiviral Res. 2022;202:105313. doi: 10.1016/j.antiviral.2022.105313 35367280

[91] DeWald LE, Dyall J, Sword JM, et al. The calcium channel blocker bepridil demonstrates efficacy in the murine model of Marburg virus disease. J Infect Dis. 2018;218(suppl_5):S588–s591. doi: 10.1093/infdis/jiy332 29982632 PMC6249584

[92] Dionicio CL, Peña F, Constantino-Jonapa LA, et al. Dengue virus induced changes in Ca(2+) homeostasis in human hepatic cells that favor the viral replicative cycle. Virus Res. 2018;245:17–28. doi: 10.1016/j.virusres.2017.11.029 29269104

